# Using Wireless Sensor Networks and Trains as Data Mules to Monitor Slab Track Infrastructures

**DOI:** 10.3390/s150715101

**Published:** 2015-06-26

**Authors:** Eduardo Cañete, Jaime Chen, Manuel Díaz, Luis Llopis, Ana Reyna, Bartolomé Rubio

**Affiliations:** Department of Languages and Computer Science, University of Málaga, Boulevar Louis Pasteur 35, Málaga 29071, Spain; E-Mails: hfc@lcc.uma.es (J.C.); mdr@lcc.uma.es (M.D.); luisll@lcc.uma.es (L.L.); reyna@lcc.uma.es (A.R.); tolo@lcc.uma.es (B.R.)

**Keywords:** wireless sensor networks, slab track infrastructure protection, communication architecture, structural health monitoring

## Abstract

Recently, slab track systems have arisen as a safer and more sustainable option for high speed railway infrastructures, compared to traditional ballasted tracks. Integrating Wireless Sensor Networks within these infrastructures can provide structural health related data that can be used to evaluate their degradation and to not only detect failures but also to predict them. The design of such systems has to deal with a scenario of large areas with inaccessible zones, where neither Internet coverage nor electricity supply is guaranteed. In this paper we propose a monitoring system for slab track systems that measures vibrations and displacements in the track. Collected data is transmitted to passing trains, which are used as data mules to upload the information to a remote control center. On arrival at the station, the data is stored in a database, which is queried by an application in order to detect and predict failures. In this paper, different communication architectures are designed and tested to select the most suitable system meeting such requirements as efficiency, low cost and data accuracy. In addition, to ensure communication between the sensing devices and the train, the communication system must take into account parameters such as train speed, antenna coverage, band and frequency.

## 1. Introduction

Recent advances in Wireless Sensor Networks (WSNs) [1,2], have taken a huge leap forward to achieve strict Quality of Service (QoS) requirements, consolidating it as a key technology for the Critical Infrastructure Protection (CIP) field [3]. Infrastructures such as power distribution and generation, nuclear power plants, railways, water distribution and management, can take advantage of WSN deployments to improve safety and detect structural health deficiencies. Proof of this is that the U.S. Department for Homeland Security stated, in the 2004 National Plan for Research and Development in Support for CIP, that one of the strategic goals was “to provide a National Common Operating Picture (COP)” for Critical Infrastructures, where the core of the systems would be an intelligent, self-monitoring, and self-healing sensor network. The Australian government, by means of the Cooperative Research Center for Security (CRC-SAFE), examined and developed solutions to security problems in CIP systems, including WSNs [4]. Finally, European projects related to CIP have also grown considerably in the last five years [5,6].

A good example of a Critical Infrastructure where WSNs can be applied is that of railways. In these structures, it is common to check the state of the structure using specialized equipment or even install a permanent monitoring system, e.g., fiber optic instrumentation with BOTDA (Brillouin Optical Time Domain Analysis) or distributed sensor instrumentation. One of the main disadvantages of these systems is their high cost, which means that they can only be installed at a few sites. Moreover, if there is no mobile coverage then data acquired by the system cannot be sent to the remote control center to be processed or analyzed. The architecture presented in this paper can be applied to any kind of railway infrastructure, but the prototype we have developed is especially tailored to be used in a slab track system. Slab tracks are currently being studied as substitutes for traditional ballasted tracks for railways due to their advantages such as a high stability, no maintenance or long life cycle but at the expense of a higher price. Our system can be used as a permanent monitoring system and considerably reduce the cost of installation and maintenance since no wiring is required. In addition, our approach can cope with the network coverage problem and tackle the transfer of large quantities of data reliably. Sensor nodes are deployed along the infrastructure, forming clusters, taking periodic readings on the structural health and sending this information to trains passing through, which are used as data mules which in turn means that no direct connectivity to the Internet is required for the WSN. Data collected by the trains are sent to a scalable time series database that is integrated in an application which analyzes the data and shows up-to-date information about the infrastructure, detects defect evolution, warns about abnormalities, and so forth.

The definition of the monitoring system was divided into two stages. The first stage was the sensor platform design. A previous paper presented the sensor platform [7] for our system. That paper described our experiences in the process to select a sensor platform which meets the system requirements. Efficient energy consumption sensors and energy harvesting systems were studied to guarantee the requirements in both the slab installation phase and the maintenance monitoring stage. The second stage which coincides with the proposal of this paper is the communication architecture definition. In this paper we propose different communication schemes and we carry out the necessary tests, meeting the system requirements, to validate the architecture.

Both real and simulated results [8] have been considered. Both papers are part of a project entitled Fastrack [9] funded by the European Regional Development fund program. The main objective of the project is the design of a new slab track system for high speed trains (faster than 250 km/h) that is environmentally and economically sustainable. In this context, a real time monitoring of the system is important to meet the project’s requirements. As part of the project, the system is currently being tested in laboratories at the Spanish government institution for the study and experimentation of public works, called CEDEX (Spanish Government Institution for the Study and Experimentation of Public Work).

It is worth remarking that in this project security aspects are not addressed as they are out of the scope of the project but we are aware of it is important to analyze them and see how they can impact in the project. To analyze security in this kind of infrastructures there exist some proposal like the one presented in [10], which is an innovative methodology to address Security, Privacy and Dependability (SPD) in the context of Embedded Systems.

The rest of the paper is organized as follows. In Section 2 related work is presented. Section 3 describes the system. Selected communication technologies are described in Section 4. Section 5 shows the strategy adopted in order to optimize energy consumption. In Section 6 the evaluation of the communication scheme and the overall system is presented. Finally, Section 7 concludes the paper.

## 2. Related Work

Many different papers present studies and deployments of WSNs to monitor railways, and other critical infrastructures, for example, bridges. A deployment of a railway bridge is presented in [11]. The WSN has a total of 8 nodes and a base station TmoteSky gathering data on the status of the bridge to detect deformations in the infrastructure when trains cross it. Accelerometer sensors are used to detect trains approaching the bridge and start the process of collecting data that remains active while the train is crossing the bridge. The network is self-organizing according to a protocol based on routing trees to be able to transmit information to the base station. Once the information has been received, the UMTS (Universal Mobile Telecommunications System) technology is used to send data to a remote control center. 

In [12], another TmoteSky WSN to collect information on the status of a railway bridge is presented. A sensor placed on trains is used as a mobile base that collects data from the sensor network as it travels through the bridge station. A tree-based routing protocol is used to transmit information to a number of leader nodes. They transmit the information collected to mobile nodes located on the train. The sampling frequency in [12] (around 20 Hz) is much lower than the one presented in this paper because the train’s speed is assumed to be low.

A WSN architecture is presented in [13] for monitoring the state of the railways. The system integrates accelerometers and ultrasonic sensors to detect wear and tear on roads. A hierarchical network topology is used so that there are multiple paths that can be used to reach the base station. This tolerance is achieved against failure nodes. The data collected by the network is merged as it is are sent to the base station through the use of fuzzy logic techniques.

Similar to the one presented in the previous paragraph is the system described in [14]. This also makes use of a hierarchical network and ultrasonics to detect possible problems in the railway’s sensors. It also introduces the use of image processing and electromagnetic detection of dangerous objects on the railway tracks.

Another architecture for the monitoring of railway infrastructure, which includes a WSN as an integral part of it, is given in [15]. The infrastructure, called SENSORAIL, integrates different kinds of sensors (such as temperature sensors and cameras) for the protection of such infrastructures. The framework provides information by means of abstractions of high-level programming. It also incorporates a threat detection system based on the information acquired by the sensors. This paper focuses on the system architecture instead of the hardware details.

In [16] the use of WSNs is proposed to control the signaling system and train control on the railway. Currently, wired networks that provide high reliability but at very high costs are used. Using WSNs would reduce costs and decrease maintenance costs. To maximize efficiency and reliability, the network is composed of heterogeneous sensors connected hierarchically. The sensors deployed in the railways make use of the IEEE 802.15.4 [17] protocol to communicate and organize themselves so that there are no redundant paths. The information collected by these nodes is transmitted to access points that use more powerful communication technologies, such as GPRS (General Packet Radio Service) or WiMAX (Worldwide Interoperability for Microwave Access), to transmit the information to the control centers.

In [18] installing permanent sensors for monitoring the condition of train bearings and detecting potential problems in them, such as locked brakes and overheating in bearings, is proposed. Two particular aspects are studied: the behavior of the wireless communication system of sensors with respect to where the sensors are installed on the train and energy harvesting techniques to minimize maintenance of the sensors. The results indicate that the radio transmitters perform better when placed above the train than when placed beneath. They also identify obtaining energy through vibrations as most promising for such applications. The integration of sensors in the wheels makes the energy harvesting easier. In our proposal, vibrations are attenuated due to the integration of nodes inside the slab track.

In [19] WSN technology is used to monitor the integrity of freight trains. Specifically, sensors are used to detect situations in which wagons become separated from the locomotive for unjustified reasons. Accelerometer and vibration sensors are able to detect whether the train is moving. They propose mechanisms for energy harvesting. However, the hardware prototype does not include any of these proposals.

In [20] the use of WSNs is proposed for early earthquake detection and control of security in railway networks. The WSN deployed in areas with high seismic activity is used to detect the onset of an earthquake with enough time to communicate the information to the control center. The latter, depending on the location of the earthquake and its severity, takes the most appropriate decision, for example, completely stop the train.

In [21] a WSN is proposed, based on ZigBee [22], for monitoring railway tracks. Specifically, distance and vibration sensors are used to report the state of the route and the presence of other trains. They propose piezoelectric sensors to detect vibrations and do not study any energy harvesting mechanism.

The proposals described in this section deal with the typical railway infrastructures based on ballast and they are deployed in critical infrastructures such as bridges. Our proposal focuses on the definition of a communication architecture for the particular characteristics of slab track installations. As stated, these facilities are more suitable for the passage of high-speed trains. In addition, the proposal describes the communication architecture for the deployment. In this paper, we detail the studies and tests used to decide the best performing communication architecture for the slab tracks. 

## 3. System Description

In this section, the complete system developed in the project is described. First, the main system requirements are detailed. Section 3.2 shows the sensor platform meeting the requirements. Next, the communication architecture is presented. Finally, we describe the main characteristics of the control center with the specialized software developed to monitor and analyze the defects of the railway infrastructure.

### 3.1. System Requirements

In order to clarify the design of the on-board components and the communication architecture of the proposed system, the set of requirements are detailed in this section. The monitoring system is based on WSNs which are able to get information about the structural health of the railway infrastructure and transmit the results to a remote control center. This system is used to increase the safety, detect issues and control the aging process of these kinds of infrastructures. The sensors integrated in the slab track have two main functions:

(1) Provide assistance in the installation phase of the slabs. The platform should provide a mechanism to configure sensors according to the terrain conditions, establishing the parameters specified during the design stage.
(2) Provide information on structural integrity of the slabs and possible displacement (inclination and distance), vibration, *etc*. during their use.

During the installation phase, sensors integrated on board will have to give the information necessary to:

(1) Calculate the distance between slabs. This distance will not exceed 1 cm with a tolerance of ±0.1 cm.
(2) Calculate the slope of the slab. It is necessary to achieve a precision of 0.1 degree.
(3) Calculate the temperature of the device. An accuracy of ±0.1 degree is enough.

During the maintenance phase, the sensors will provide the information necessary to ensure the real-time knowledge of the state of the infrastructure:

(1) Acceleration readings of the slab tracks when trains are passing over them. In order to detect defects or abnormal situations, the frequency of the different vibrations obtained from the acceleration data is studied. To do this the raw acceleration data are transformed to the frequency domain by means of the Fast Fourier Transform (FFT). Due to the vibrating nature of slab tracks, acceleration must be obtained with a 0.005 g resolution, accuracy of 15% and bandwidth of 1 Hz–800 Hz.
(2) Displacement of the slab relative to its original position recorded during the installation phase.
(3) Alarms for low battery and sensor failure.

### 3.2. Sensor Platform

**Figure 1:**
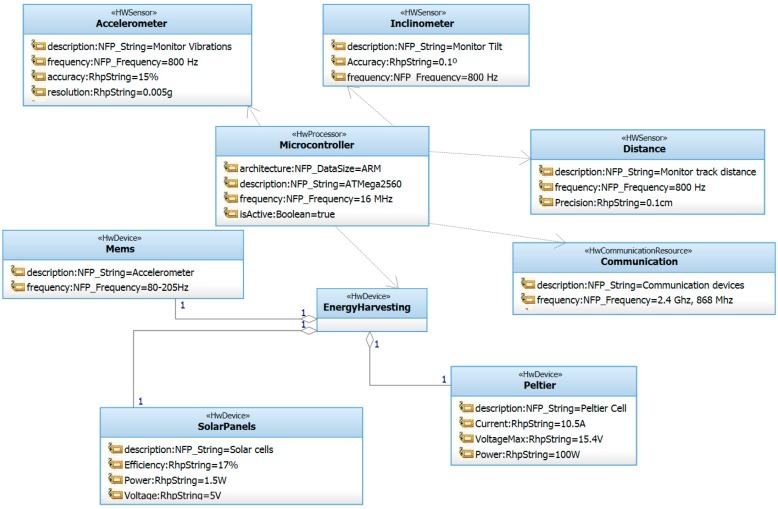
Hardware architecture.

In order to meet the requirements presented in the previous section, Figure 1 shows the high level architecture for the hardware components of the system. It is composed of a microcontroller that controls different sensors. The system is powered by a battery and optionally by an energy harvesting module. The module is installed inside selected slab tracks to monitor different parameters as described in this section.

The most viable option for the system prototype both from an economic point of view and for flexibility reasons was the use of the open-source computing platform known as Arduino. This platform offers a good balance between capabilities and energy consumption at a reasonable price. The MEGA 2560 model has been chosen. It provides more than one serial port which is necessary for some of the sensors selected. The platform integrates the ADXL345 accelerometer sensor, the SCA100T-D2 inclinometer sensor and the SRF05 distance sensor. In addition, a 1.5 W solar panel has been used as the energy harvesting mechanism in order to increase the life time of the battery that supplies energy to the board. Finally, the sensor platform has been equipped with the Xbee communication modules described in the following sections. A complete description of our selected sensor platform can be consulted in [7].

### 3.3. Communication Architecture

**Figure 2:**
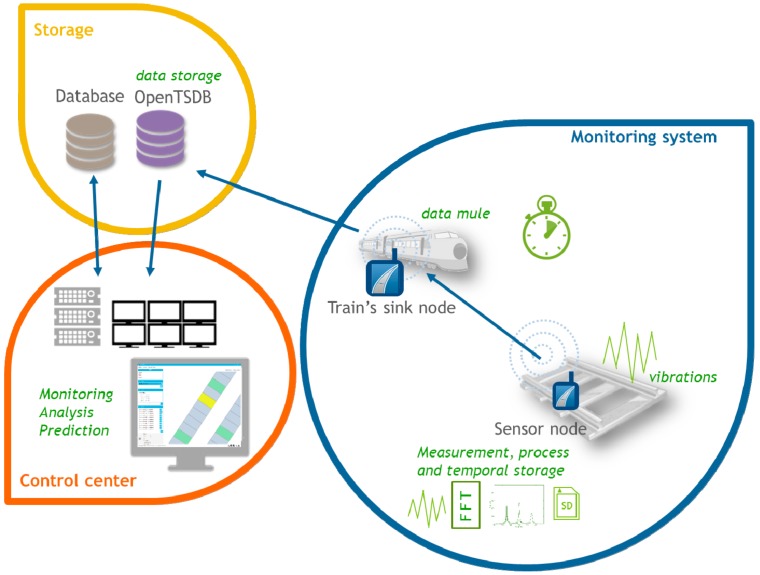
Maintenance phase communication architecture.

Our sensor platform, as depicted in Figure 2, is designed to be part of the slab track, inserted before its installation, so that it can be used in both installation and maintenance phases. The slab track therefore becomes an active element capable of monitoring and reporting information about the environment such as vibration performance, distance and inclination and is also able to assist operators in the installation phase. The number of monitored slab tracks is based on factors such as the cost of the monitoring system or the criticality of the railway section.

Railway infrastructures cover really large areas, possibly in places where there is no Internet coverage or permanent electricity supply. The system proposed in this paper makes use of autonomous sensor nodes powered by batteries and solar panels. They wake up periodically to gather information. In order to deal with the connectivity/coverage issue the sensor nodes use trains as data mules. The information collected by the sensors, after being partly analyzed and reduced, is received by sink nodes placed on each of the passing trains. Finally, trains send the information to the remote control center.

Most of the normal vibrations produced in the slab track are located in the 1–800 Hz range. In order to capture this acceleration data a high sampling rate is necessary. However, sensors are energy-limited devices and their communication speed is relatively low and energy consuming. In order to avoid transmitting a huge amount of data from each sensor to the train, each sensor locally processes the acceleration and obtains vibration frequency peaks in the frequency domain. The algorithm carried out locally by each sensor is as follows:

(1) Each sensor collects triaxial raw acceleration when a train is passing.
(2) Acceleration data is transformed to the frequency domain using the FFT for each window.
(3) The highest peaks in acceleration in the frequency domain are detected (in all windows).
(4) The three biggest peaks (amplitude/frequency pair) that correspond to the main frequencies at which the slab track vibrates and their corresponding timestamps are sent to the next train that passes by and from the train to the control center (Figure 2).

Additionally, periodic readings of temperature, inclination and distance between slab tracks are collected and stored. All these three magnitudes are measured with different sensors installed in the sensor nodes. To measure inclination dedicated inclinometers have been installed whereas to measure distance, ultrasonic sensors have been used. They are used to detect changes in the initial conditions of the deployment and therefore detect possible issues or abnormal situations. More details about the selection process and integration of these sensors are explained in [7].

**Figure 3:**
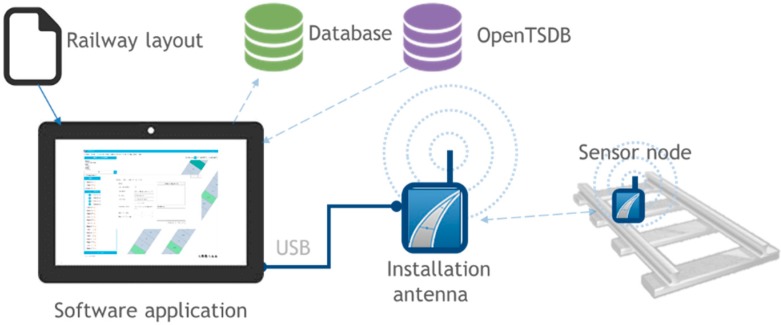
Installation phase communication architecture.

The communication architecture described allows us to monitor the structural health of the slab track. In addition, it can also be used to help in the installation phase (Figure 3). During the installation, sensors have to provide their initial measurements as they are needed as reference values for the analysis. Sensors also have to give their unique identifiers to the application so they can be stored with their geographical position. 

### 3.4. Centralized Control System

**Figure 4:**
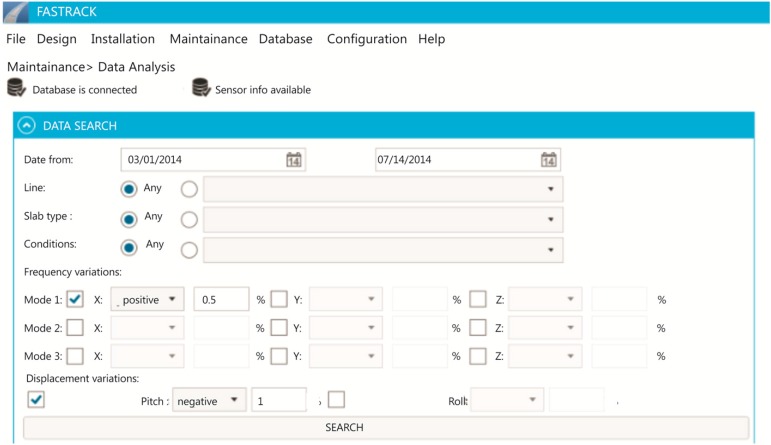
Control Center: Data analysis.

The last component of the system is the control center where the data collected by the trains from the slab tracks is stored. An Open TSDB (Time Series Database) has been integrated in the system from which it is possible to query data from the slab track nodes to define patterns of defects or enable warnings. Figure 4 shows how it is possible to define filters to analyze the data extracted from the slabs.

**Figure 5:**
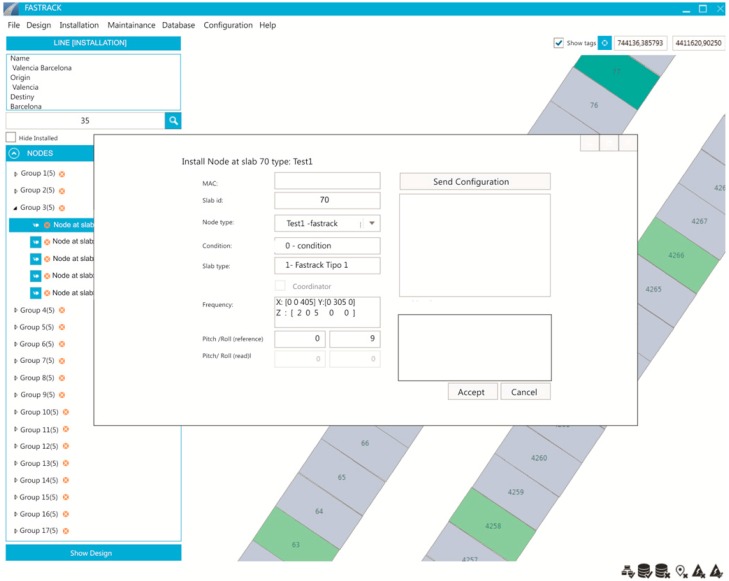
Control center: monitoring of tracks.

**Figure 6:**
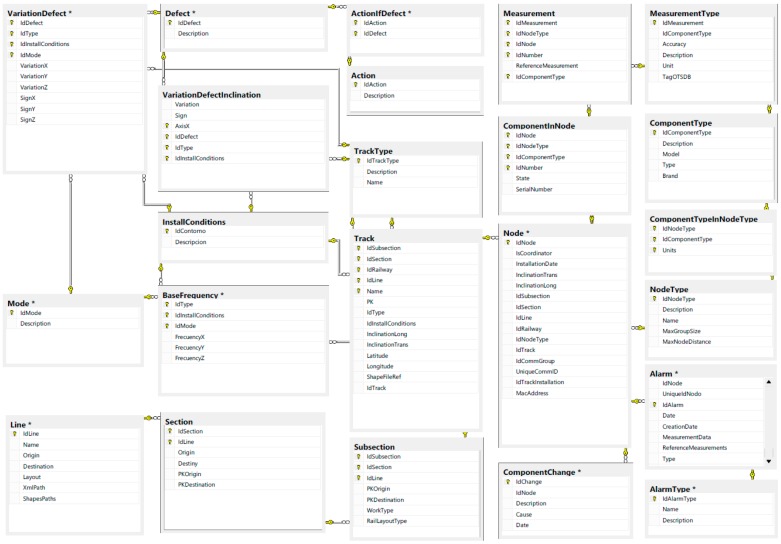
Database entity relationship diagram.

The centralized control system includes the geographical information that indicates in which slabs the nodes will be installed. A relational database has also been integrated to store this information. Figure 6 shows the database diagram. Figure 5 shows a track section which indicates where the nodes are installed. Additionally, it is possible to get their configuration. 

## 4. Communication Technologies

As stated, trains will be responsible for collecting the information generated by the monitoring system. It is therefore necessary to study the different options to communicate the deployed WSN in the slab track with passing trains. Additionally, the network organization also has to be considered. For this task we have studied two main technologies: Radio transmitters and RFID (Radio-frequency identification). A third option, based on GPRS is briefly presented for the case where data mules are not available. Energy efficiency, the highest range with the least cost and fault tolerance are essential requirements to meet in the different architectures. The following subsections describe these alternatives of communication architecture. Due to cost limitations, GPRS and RFID communication architectures were not tested, but we think the study carried out could be useful.

The knowledge of how much information can be sent to each of the data mule trains is essential to determine the feasibility of this system. This depends on two main factors:

(1) The train speed: Higher speed less data to communicate.
(2) The antenna range of the radio transceivers in the train and slabs: Higher range, higher communication bandwidth.

As an example, Table 1 shows the time that two radio transmitters (in the train and the slab track) are in range. In this example, we consider that both antennas have the same range and that the coverage is ideal.

In a real scenario, the time each antenna has to transmit information to the train is determined by several factors such as the distance between radio transmitters on the track, train speed, amount of information you want to convey, etc. Let us note that at 320 km/h the time that the antennas remain in range is very small. Therefore, we must be sure that the communication is feasible and how it affects the rate of these results in a real scenario. Table 2 shows a summary of the different architectures presented in this section.

**Table 1:** Time (s) two antennas can communicate assuming one of them is placed in a train.

		Train Speed(km/h)						
		50	100	150	200	250	300	320
**Antenna Range (m)**	50	7.2	3.6	2.4	1.8	1.44	1.2	1.125
	100	14.4	7.2	4.8	3.6	2.88	2.4	2.25
	150	21.6	10.8	7.2	5.4	4.32	3.6	3.375
	200	28.8	14.4	9.6	7.2	5.76	4.8	4.5
	250	36	18	12	9	7.2	6	5.625
	300	43.2	21.6	14.4	10.8	8.64	7.2	6.75
	350	50.4	25.2	16.8	12.6	10.08	8.4	7.875
	400	57.6	28.8	19.2	14.4	11.52	9.6	9
	450	64.8	32.4	21.6	16.2	12.96	10.8	10.125
	500	72	36	24	18	14.4	12	11.25
	550	79.2	39.6	26.4	19.8	15.84	13.2	12.375
	600	86.4	43.2	28.8	21.6	17.28	14.4	13.5

**Table 2:** Proposed communication architectures.

	ZigBee and 868 MHz Radios Using Groups	868 MHz Radio Using Groups	868 MHz Radio (Independent)	RFID
**Group organization**	Yes	Yes	No	No
**Direct communication (Installation mode) **	No (only by means of coordinator)	Yes	Yes	Yes (operator need to be close to the RFID tag)
**Communication interference node-train**	Medium	Medium	High	No
**Price**	Low	Medium	Medium	Medium (RFID readers are expensive)
**Hardware**	Digi Xbee-PRO ZB Digi Xbee 868 MHz (S5)	Digi Xbee 868 MHz (S5)	Digi Xbee 868 MHz(S5)	Not tested
**Power consumption**	Medium	Medium-High	High	Low
**Number of radio modules in coordinator**	2	1		
**Interference among nodes (different groups)**	No (different channels)	Low		

### 4.1. ZigBee and 868 MHz Radio Modules Using Groups

**Figure 7:**
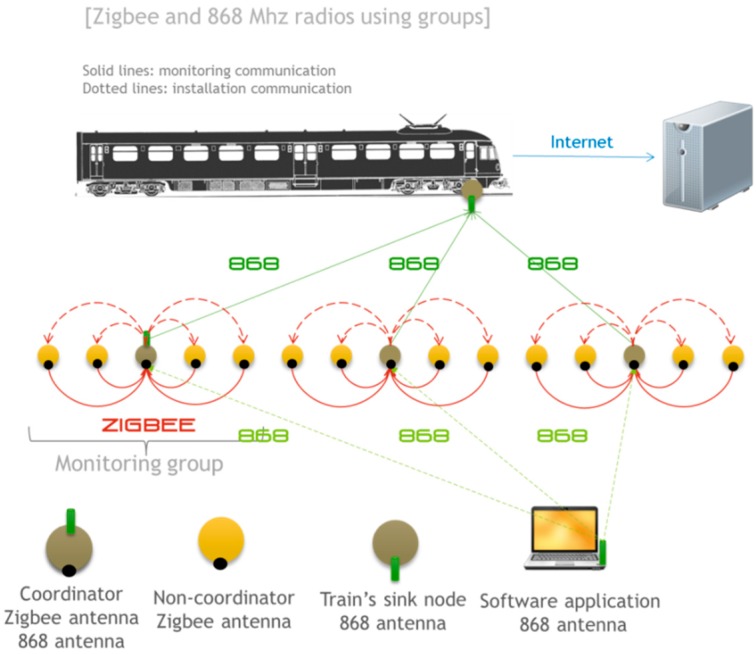
ZigBee and 868 MHz radios using groups: Communication architecture.

The first of the proposed architectures for the monitoring system uses XBee ZB PRO S2 and 868 modules. Figure 7 shows an overview of this architecture. The nodes deployed in the railway are divided into small monitoring groups. The size of these groups is chosen based on factors such as distance between monitored slab tracks, radio antenna range, interferences. Each node of the monitoring group uses ZigBee to send the information to a single coordinator who manages the group. The coordinator stores this information temporarily until a train passes. As a train is detected, the 868 MHz module transmits the information to the train. ZigBee uses the 2.4 GHz band so that transmissions are faster and more reliable but at the expense of a lower range. On the other hand, the 868 MHz communication has a larger range and does not require an association protocol between nodes, which suits the communication requirements between the train and the sensor nodes.

Note that each node with a ZigBee radio in the monitoring group, except the coordinator, is configured as “end device”, as defined in the ZigBee standard, which allows them to sleep to preserve battery, but does not permit multi-hop routing. This is the reason why we have to communicate directly with the leader of the group and cannot use multi-hop routing (due to restrictions in those devices an end-device cannot route information). The group leader or coordinator must therefore be configured as ZigBee coordinator as a necessity. Each coordinator, and thus each group, has a different PANID (Personal Area Network Identifier). This way, the communication between nodes in different groups is not allowed and, what is more important, there is no interference between adjacent monitoring groups because they operate in different radio channels. The coordinator nodes are susceptible to interference between each other when they send information to the train and use the same channel.

### 4.2. 868 MHz Radio Modules Using Groups

**Figure 8:**
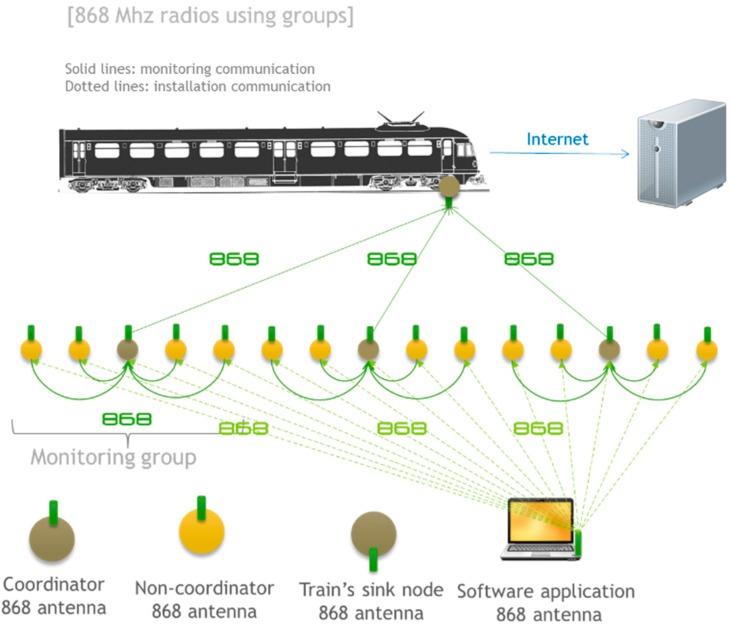
868 MHz radios using groups: communication architecture.

The next architecture, shown in Figure 8, is based on the use of 868 MHz radio modules. The architecture is composed of groups controlled by a coordinator. Since the communication between 868 MHz modules does not require association, the same communication module can be used to communicate with the train and other nodes in the same monitoring group. However, there can be interferences between the groups and trains, especially if the monitoring group is densely deployed. To avoid this, during the transmission of information from the coordinator to the train, the remaining 868 modules in the group are set to sleep. The coordinator nodes are susceptible to interference between each other when they send information to the train and use the same channel.

### 4.3. 868 MHz Radios without Using Groups

**Figure 9:**
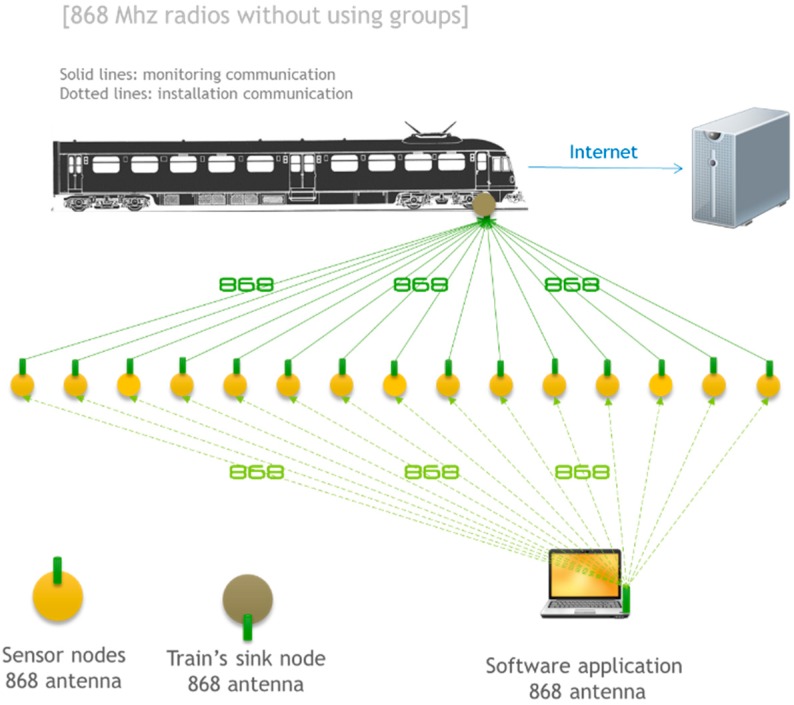
868 MHz radios without using groups.

An alternative communication architecture based on 868 MHz radio modules is presented in Figure 9. This option is similar to the previous one in terms of hardware. However, there are no monitoring groups in this architecture. That is, all nodes act completely autonomously. Each node is responsible for sending the information to the train without the need for a coordinator. The communication between the operator and each node also takes place directly. This mode is especially susceptible to interference because there is only one transmission channel and neighbor nodes will broadcast information to the train in the same time instant. However, in terms of flexibility this is the best option since all nodes are independent from each other and there is no association or group management protocol, which saves energy.

### 4.4. RFID Based 

Another possibility of communication between the nodes of the monitoring system and trains is using RFID tags. Broadly speaking, RFID is a wireless technology for short range and low power consumption that has two components: RFID tags and RFID readers. An RFID tag can store and send information to a reader via radio waves. The tag consists of an antenna, a wireless transducer and a microchip to store data obtained from sensors. Moreover, the reader is able to automatically read data recorded on the tag when it is physically close to it (in the range of centimeters or even meters).

**Figure 10:**
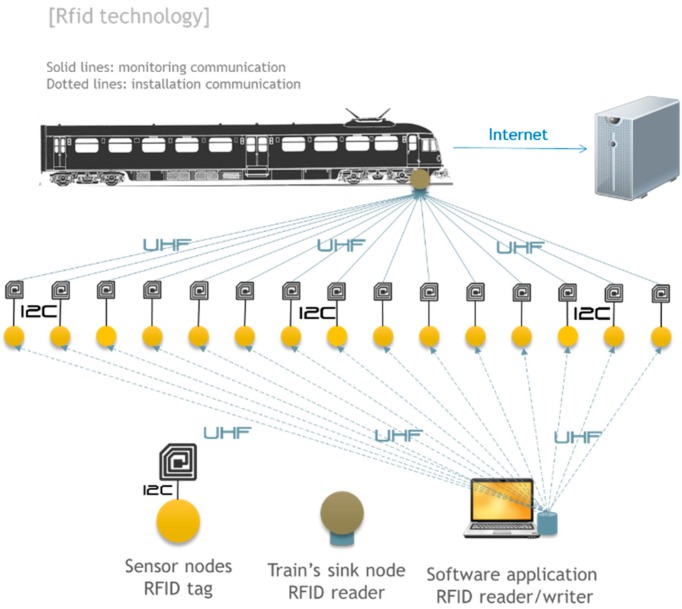
RFID: communication architecture.

In this proposed architecture, shown in Figure 10, the data will be read by the train at no additional cost to the energy monitoring system since RFID tags are typically passive elements. The RFID reader is therefore the element that is installed on the train and passes over each of the RFID tags deployed in the slab track to collect the information. The Arduino over which the system has been built needs a way to record data on the RFID tag prior to being read by the train. One proposal for this is using the Monza chip from the company Impinj [23]. These RFID chips, in addition to their normal function, can be read and written via an I2C line. This line would be used by the Arduino to program the tags with data from the sensors.

In order to analyze the feasibility of this option it is necessary to take into account the maximum distance at which you can read the labels and the speed at which you can perform the reading. Other factors to consider in this feasibility analysis are:

(1) Consumption of I2C protocol for recording data.
(2) Read speed.
(3) Maximum reading distance.
(4) Device capability.

This architecture was discarded due to the high costs of the initial design and manufacturing of the tags. However, we believe that, if a high number of tags were manufactured and deployed, this solution would be feasible.

### 4.5. GPRS Communication

**Figure 11:**
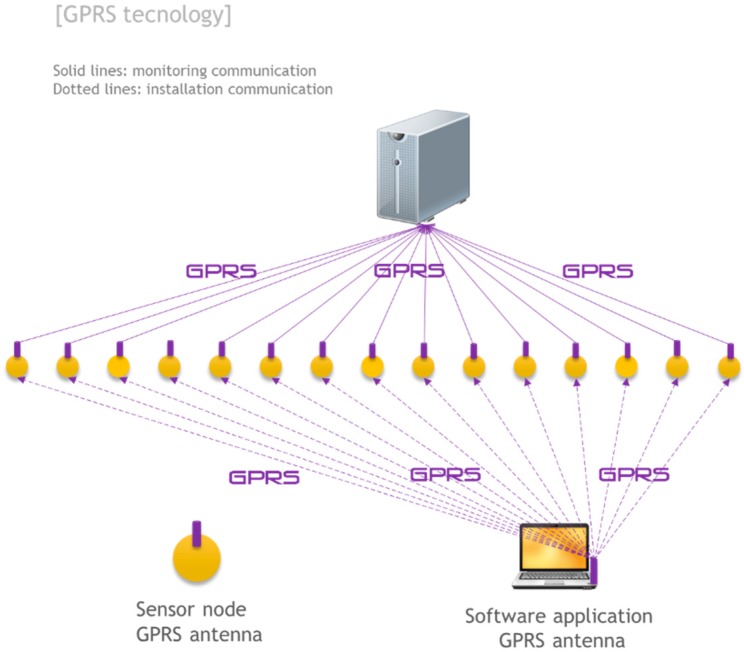
RFID: communication architecture.

A communication option that does not use trains as base stations is the use of communication technologies based on cellular networks, such as GPRS or more concretely the GSM-R (Global System for Mobile Communications–Railway) [24] protocol, specially tailored for railway communications. GPRS is a protocol for packet-oriented mobile communication and operates on the known telephone networks and 2G and 3G data. In this architecture, each Arduino would work independently and would have a GPRS device that allows sending information directly to the Internet without having to communicate with each other or rely on trains (see Figure 11).

The main characteristics that must be addressed are the amount of transmitted data for battery consumption and the cell coverage. This option is not viable where there is no cell coverage. These two limitations do not make GPRS the most attractive option. However, due to its robustness and availability (by not relying on trains to communicate) it is an option that may be appropriate to take into account in specific sections of slab tracks or critical sections where continuous monitoring, quick response and high reliability are required.

## 5. Energy Consumption Optimization Strategy

One of the main requirements of the project is to ensure the system works for as long as possible. Therefore, the node is designed to be sleeping (consuming only several µA) almost all the time. That is, the node will wake up only when the train is approaching to be able to perform two key tasks:

(1) Enable accelerometers to collect data from vibrations produced by the approaching train.
(2) Transmit data collected during the passing of the next train.

To go from the state asleep to awake, the node needs an external interrupt that alerts the node to the approaching train. In the following sections two solutions are designed to awake the node in time to perform the two aforementioned tasks.

### 5.1. Detection Using the Accelerometer in the Monitoring Node

The accelerometer installed on the node detects when the train is passing overhead and it is responsible for activating the micro-controller to record data or to send the data to a train. 

This solution has the following disadvantages:

(1) Depending on the nature of the railway infrastructure and where the sensor is deployed, the vibration response of the slab track may be different. It would be necessary to study the different vibration patterns in the slab tracks.
(2) If the communication between antennas requires association there is a certain amount of time that nodes cannot communicate with the train. The use of different types of antennas (e.g., directional antennas) could help to extend the communication range and therefore tackle this problem.

### 5.2. Detection Using Time Scheduling

This project is focused on monitoring high-speed trains which usually follow strict timetables. That is, each train will pass over the top of each slab track at the same time every day. This feature can be exploited to use a clock to wake up the nodes at the scheduled time with a margin of ±10 min. During that time, the antennas will wait for “beacon signals” from the train. Upon detecting a “beacon signal”, nodes will begin to record the data collected by the accelerometer. 

Disadvantages of this solution:

(1) The nodes would be active longer than the previous option.
(2) If the train schedule changes, nodes have to be reprogrammed (it can be done over-the-air by the operator).
(3) An antenna continuously emitting “beacon signals” needs to be placed on the train.

## 6. System Evaluation

This section details the different tests carried out to analyze the architectures proposed. The tests are divided into three different sections. Section 6.1 and Section 6.2 detail individual tests with the Xbee ZB S2 and Xbee 868 radio modules, respectively. The complete system is tested in Section 6.3.

### 6.1. Xbee ZB PRO S2 Radio Module 

As discussed, one of the proposed architecture uses an XBee ZB PRO S2 module for communication within the monitoring group. This module supports the ZigBee protocol using the 2.4 GHz free band. To study the feasibility of this module, several tests have been carried out.

#### 6.1.1. Association Time

As specified in the ZigBee standard, a conventional ZigBee network is divided into different clusters. Each cluster is controlled by a unique “coordinator” which controls the cluster and allows other nodes to become part of it. A node which wishes to join the group has to exchange information with the coordinator to manage the joining process. We measured the average time a ZigBee node takes to join an existing group and the results show that the time varies from about 3 to 5 s. This association process is necessary for a node to be able to communicate with other nodes. In other words, when a ZigBee coordinator and another node that wants to join the group are in range the sensor must wait a minimum of 3–5 s before being able to send information.

#### 6.1.2. Range Test

In this test the maximum range of the Xbee PRO S2 has been tested. Different combinations of antennas for the receiver and the transmitter were tested ranging from 2 dBi to 5 dBi. The test consists of measuring the maximum distance at which two different nodes can communicate. Table 3 shows the results obtained. The tests performed in this section assume that the two radio modules are already associated. The antennas have been placed at different heights (on the floor and at a height of 2 m).

**Table 3:** Maximum communication range of Xbee PRO S2.

Transmitter		Receiver		Distance
Antenna Gain	Height	Antenna Gain	Height	
Small (2 dBi)	Floor	Small (2 dBi)	Floor	60 m
Small (2 dBi)	2 m	Small (2 dBi)	2 m	200 m
Small (2 dBi)	2 m	Small (2 dBi)	2 m	450 m
Big (5 dBi)	Floor	Big (5 dBi)	Floor	600 m
Big (5 dBi)	2 m	Big (5 dBi)	2 m	4000 m

The results show that there is a big difference in communication range depending on where the antenna is placed. When placed on the floor, the performance of the radio module decreases significantly. For example, in the test where the 2 dBi antenna has been used, both in the transmitter and the receiver, the communication range is 60 m. This is much less than the range depicted in the datasheet (hundreds of meters).

#### 6.1.3. Transmission and Mobility Tests

**Figure 12:**
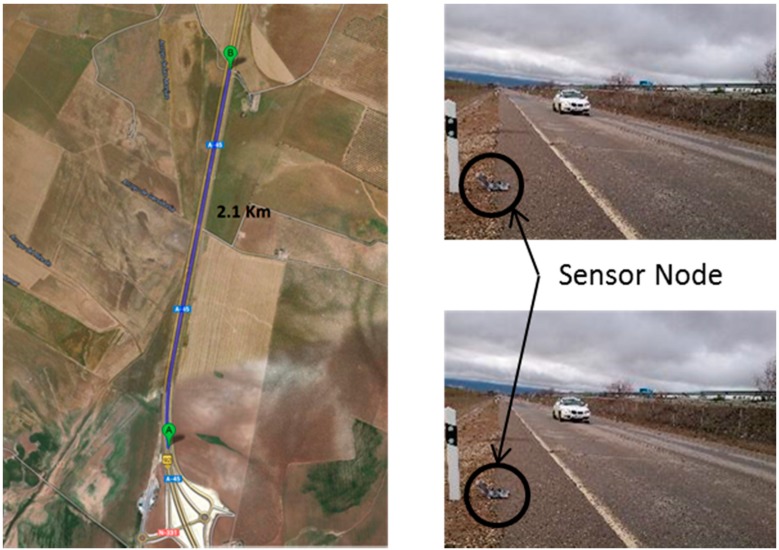
Evaluation scenario for the range tests.

The goal of the mobility tests is to measure the amount of data that two radio modules are capable of exchanging when one of them is moving. This test simulates the scenario where the sensor node deployed in the railway infrastructure (static node) sends information to a radio module located on the train (mobile node). Tests simulate the train with a car driving at a controlled speed. The vehicle drives along a straight road with a length of 2.1 km, as shown in Figure 12. The sensor node located on the floor continuously transmits information to the car. The car starts from a location where the radios are out of range, passes next to the sensor node on the floor and continues until the radios are again out of range. An antenna of 2 dBi has been used for the receiver/transmitter respectively.

**Figure 13:**
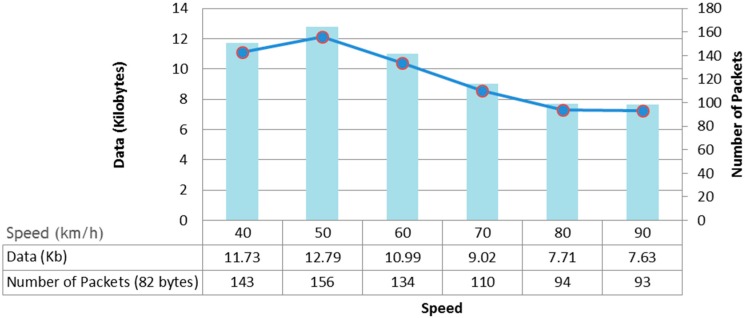
Results of the transmission and mobility tests (2.4 GHz and 2 dBi antennas).

Figure 13 shows a graph generated from the data obtained, varying the speed of the vehicle. At a speed of 80 km/h, the data received is less than 8 Kb. This amount of data is relatively low and is partly due to the fact that these modules require a long association time of about 5 s. Given that a high-speed train reaches speeds of over 300 km/h, these modules are clearly not suitable for node-to-train communication.

However, these modules have a good performance under static conditions at a competitive price compared to the Xbee 868 modules. This module is, therefore, a good candidate for the static nodes deployed in the railway infrastructure (as proposed in Section 4.1).

### 6.2. Xbee 868 MHz Radio Module

According to the results of the ZigBee communication tests, another alternative must be considered to ensure a more powerful node-to-train communication. The XBee 868 radio modules have been tested. According to the manufacturer, they allow the use of high gain antennas, reaching a range of up to 80 km.

#### 6.2.1. Range Test

As done in Section 6.1.2 the maximum range of the Xbee 868 radio module was tested. The tests were conducted with two different antennas: a 0 dBi (referred to as small) and a 4.5 dBi (referred to as big). Different combinations of large and small antenna for the receiver and the transmitter were tested (Table 4).

**Figure 14:**
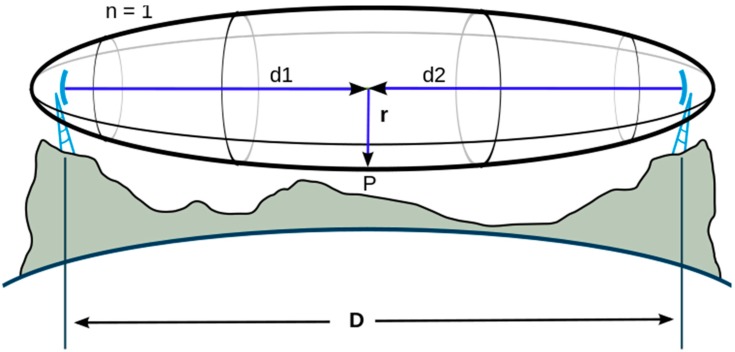
Fresnel zone.

The communication distance of 868 modules is not as high as expected. It is about 600 m in the best case (using big antennas), while in the specification sheet the antenna is said to have a range of tens of kilometers. However, this range is only achievable when using big directional antennas and only if the Fresnel zone (see Figure 14) is respected. Due to the project’s requirements (space reasons), we are restricted to using small antennas and the transmitting antenna must be installed at ground level, the reason why a high percentage of the Fresnel zone is obstructed by the ground.

**Table 4:** Maximum communication range of Xbee 868 MHz.

Transmitter		Receiver		Distance
Antenna Gain	Height	Antenna Gain	Height	
Small (0 dBi)	Floor	Small (0 dBi)	Floor	110 m
Small (0 dBi)	Floor	Small (0 dBi)	2 m	150 m
Small (0 dBi)	2 m	Small (0 dBi)	2 m	300 m
Big (4.5 dBi)	Floor	Big (4.5 dBi)	2 m	200 m
Big (4.5 dBi)	2 m	Big (4.5 dBi)	2 m	600 m

#### 6.2.2. Transmission and Mobility Tests

**Figure 15:**
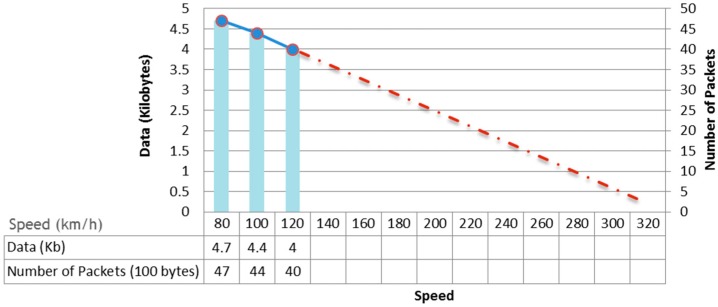
Results of the transmission and mobility (868 MHz and 0 dBi antennas).

The goal of the speed tests is to measure the amount of data that two radio modules are capable of exchanging when one of them is mobile. The same scenario as presented in Section 6.1.3 has been used. Table 5 and Figure 15 show the results obtained at low velocity (80, 100 and 120 km/h). Data for higher speed values has been extrapolated. These results should be taken with caution and require verification in real tests.

Based on linear extrapolation using 0 dbi antennas, a single transmitter would be able to transmit about 5 100-byte packets to a train moving at 320 km/h. Thus, if we assume an average of 5 nodes in range with each other communicating with the train and knowing the physical medium for communication is shared, we cannot ensure that all nodes can send information to each train. It is therefore necessary to use additional mechanisms to increase the effective bandwidth of the radios that send information to the train. Possible solutions to this problem are the use of multiple radio receivers on the train, for example one at the front and one at the back, the use of more powerful antennas or the use of more sophisticated channel access methods such as TDMA (Time Division Multiple Access).

**Table 5:** Transmission and mobility tests with different antennas (868 MHz).

	Small Antenna (0 dBi)		Big Antenna (4.5 dBi)		
**Km/h**	Packets	Bytes		Packets	Bytes
**80**	47	4700		154	15,400
**100**	44	4400		145	14,500
**120**	40	4000		131	13,100

In order to study the possible interferences caused by the use of multiple antennas in the same area (as in a real environment), an additional test was carried out. We added a second fixed emitter. This test was performed with a constant speed of 100 km/h, with both 2 and 3 emitters. Table 6 and Table 7 summarize the results.

**Table 6:** Transmission and mobility tests with two transmitters (868 MHz, 0 dBi).

	Packet Sent		
	Packet	Kilobytes
**Transmitter 1**	16	1600
**Transmitter 2**	26	2600
**Total**	42	4200

**Table 7:** Transmission and mobility tests with three transmitters (868 MHz, 0 dBi).

	Packet Sent		
	Packet	Kilobytes
**Transmitter 1**	13	1300
**Transmitter 2**	13	1300
**Transmitter 3**	24	2400
**Total**	50	5000

If multiple transmitting antennas are in range, the amount of data that can be transmitted with each of them is relatively low. This behavior is to be expected since a shared medium is being used and nodes cannot transmit simultaneously. However, the amount of information that can be sent by each radio module is not the same, depending as it does on the time at which each antenna starts sending or coming into range, which is something that cannot be controlled. For example, in some cases where we have used two emitters we noticed that one of them is capable of transmitting much more information than the other one.

### 6.3. Complete System

To assess the feasibility of the monitoring system, the proposed architecture described in Section 4.1 has been implemented and tested in-lab. Out of the three architectures based on radio transmitters presented, the one that combines two different modules has been chosen because of the following advantages:

(1) 868 MHz radio modules do not require association so they are more suitable for node-to-train communication.
(2) 2.4 GHz communication is more reliable in short range communications and has a lower cost and power consumption so it is more suitable for node to node communication (in the railway infrastructure).
(3) There is no interference between node-to-node and node-to-train communication.

This architecture divides the nodes deployed in the railway infrastructure into different groups. The information generated in each group is collected by a coordinator node using ZigBee in the 2.4 GHz band (XBee PRO S2 radio module). Each time a train passes, the coordinator of each group uses the 868 MHz band (XBee 868 radio module) to send information to a receiver node installed on that train. We assume that the train has Internet connectivity. Therefore, once the information has been received on the train it will be sent to a remote database for storage.

**Figure 16:**
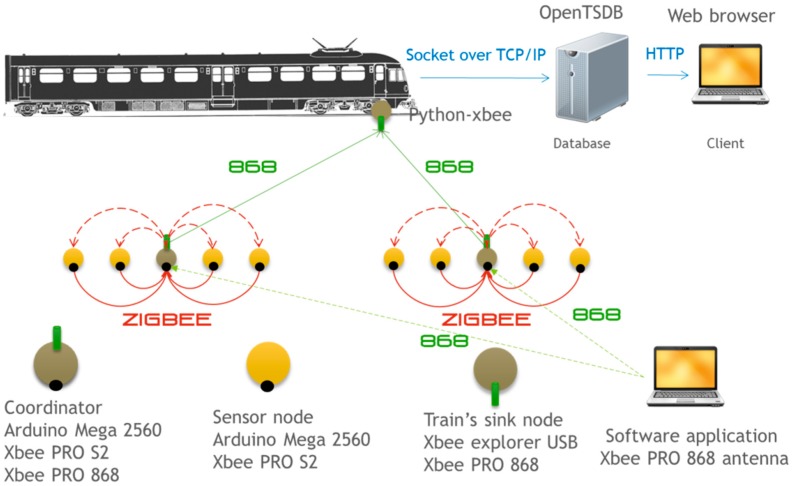
Complete system communication architecture.

Figure 16 shows the overall system architecture. A set of nodes marked with circles are displayed. These nodes are organized into groups with a single coordinator. The group coordinator receives the information collected by other nodes using ZigBee. Coordinators then detect when a train is approaching and using point to point communication in the 868 MHz band send the information to the train.

As stated in Section 3, once the train receives information it is sent to a remote database using the Internet (TCP/IP). Collecting information on the train and sending the data to the database is performed by a Python script that uses the Python-class library Xbee. The data is stored in a time series database called OpenTSDB [25]. This database has been especially optimized for temporal data like that collected by the monitoring system. Once the data is in the database, it can be accessed from any browser accessing the IP of the computer where the OpenTSDB server is deployed.

**Figure 17:**
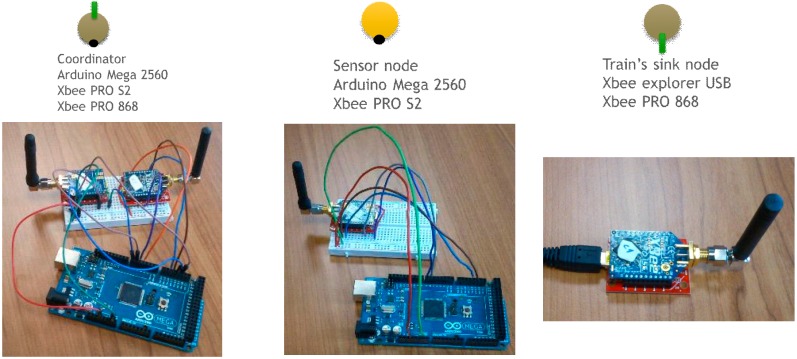
Devices of the monitoring system.

**Figure 18:**
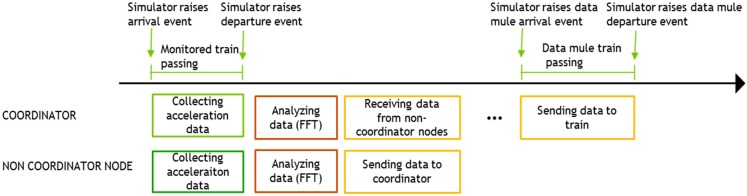
Node activity schedule.

Figure 17 illustrates the appearance of the devices used to implement the communication system prototype. For a realistic simulation we have implemented a simple train simulator. This simulator simulates the passing of a train every half an hour. The train passing by lasts 20 s which means that 20 s of acceleration data at 800 Hz will be collected. Once the data has been collected it is analyzed with the algorithm mentioned in Section 3.3. Once the peaks in the frequency domain have been identified, this information is sent to the coordinator of the monitoring group. Finally, when a data mule train is detected, all this information is sent to it. Figure 18 summarizes the schedule of the various activities that occur in the sensors with respect to the schedule of the trains.

Finally, as the data is received by the radio module located on the train, a socket connection opens to communicate with the remote server containing the OpenTSDB database. This database can be accessed from a conventional browser from the Internet. 

**Figure 19:**
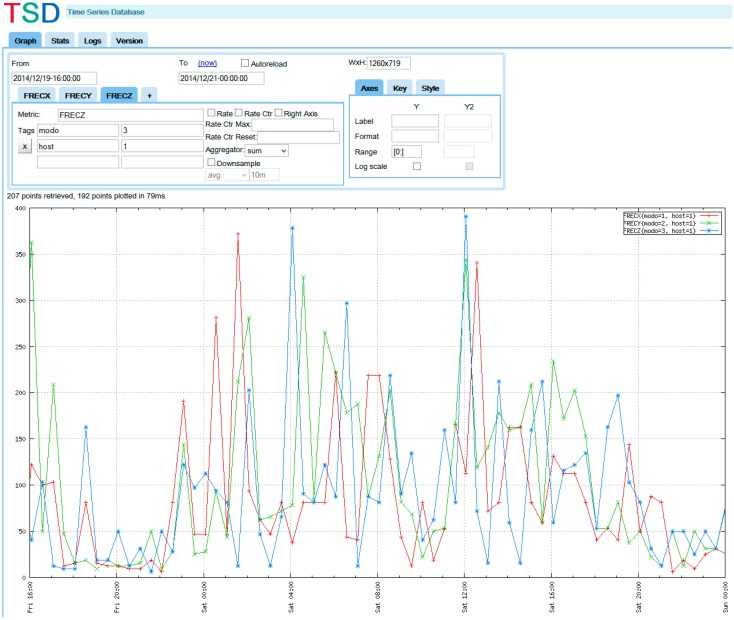
OpenTSDB screenshot with data from the test.

The test scenario has a total of two monitoring groups each of them with 3 nodes (including the coordinator) and does not take mobility into account. The test has been simulated for 2 days. In other words, this means that information from a total of 96 trains has been collected. All the train information has been successfully received at the train radio receiver and relayed to the database, which means 100% reliability has been achieved. Figure 19 shows a screenshot of a data stored in the database and queried from a browser. 

## 7. Conclusions

A monitoring system for slab track infrastructures has been presented. It provides structural health related data that can be used to evaluate their degradation, detecting and predicting possible failures. Our monitoring system, based on the use of Wireless Sensor Networks, can be used as a permanent infrastructure and considerably reduce the cost of installation and maintenance since no wiring is required. In addition, trains are used as data mules so that no direct connectivity to the Internet is necessary, solving the network coverage problem and tackling the transfer of large quantities of data reliably.

Different communication architectures have been designed and tested to select the most suitable system meeting such requirements as efficiency, low cost and data accuracy. The selected architecture divides the nodes deployed in the railway infrastructure into different groups. The information generated in each group is collected by a coordinator node using ZigBee in the 2.4 GHz band. Each time a train passes, the coordinator of each group uses the 868 MHz band to send information to a receiver node installed on that train.

The approach has been implemented, simulated and tested in-lab, and different results have been shown in this paper. We are currently involved in the deployment of the system along a real section of railway infrastructure (we still need to get the corresponding permission from the authorities) and using the data mule based communication mechanism described, we hope to achieve a real WSNs-based monitoring system for Railway Infrastructure Protection.
